# Editorial: Enhancing veterinary access through One Health and interprofessional collaborations

**DOI:** 10.3389/fvets.2026.1812413

**Published:** 2026-03-11

**Authors:** Michael J. Blackwell, T. Fisher, Jennifer Weisent

**Affiliations:** 1 Program for Pet Health Equity, Center for Behavioral Health Research, College of Social Work, The University of Tennessee, Knoxville, Knoxville, TN, United States; 2 College of Veterinary Medicine, The University of Tennessee, Knoxville, Knoxville, TN, United States

**Keywords:** access to veterinary care, community engagement, cultural competence, equity, financial accessibility, interprofessional collaboration, One Health, workforce development

Over the past decade, access to veterinary care has shifted from being framed primarily as a matter of individual client circumstance or clinic capacity to being recognized as a complex, systems-level challenge. As Topic Editors working across community-based veterinary and animal welfare initiatives, public health, education, and interprofessional partnerships, we have consistently observed that whether pets and their families are able to obtain care is shaped less by isolated clinical decisions than by the alignment—or misalignment—of social services, financial structures, workforce preparation, and community trust. These realities were a central motivation for convening this Research Topic.

Financial insecurity, workforce shortages, transportation barriers, housing instability, and historical marginalization all influence whether veterinary services are accessible and sustainable. These forces situate veterinary access within the broader landscape of public and community health, where outcomes are produced not by any single profession or sector but by interconnected systems. The Research Topic *enhancing veterinary access through one health and interprofessional collaborations* was developed to reflect and advance this evolving understanding. The articles collected here move beyond deficit-based narratives and isolated interventions to examine how veterinary access can be strengthened through interprofessional practice, community-engaged partnerships, workforce development, and structural innovation. Grounded in One Health as a care-systems framework, this body of work emphasizes that pet health, human wellbeing, and community conditions are shaped by shared social and structural determinants and that coordinated, equity-oriented strategies are required to address persistent gaps in care.

A central theme across this Research Topic is the role of interprofessional and relational care models in expanding access to veterinary services. In *One health and human animal-bond intervention strategies- assessing veterinary-social service collaborations*, the authors examine integrated service approaches that bring veterinary professionals together with social workers and community-based organizations serving unhoused and socially marginalized populations. By centering the human–animal bond and adopting a relational, trust-building framework, this work demonstrates how interprofessional collaboration can reduce access barriers, improve engagement, and support families who are often excluded from traditional veterinary systems. These findings underscore the importance of care models that treat social context not as peripheral but as integral to clinical effectiveness (Orchard, Scarbrough et al.).

Workforce development and education emerge as another critical pillar. *Beyond clinical skills: student-reported impacts of a veterinary public health externship in rural Alaska* explores how immersive, community-based training experiences shape veterinary students' professional identities and competencies. Participants reported growth not only in clinical and public health skills but also in cultural humility, communication, and systems-level thinking—capacities essential for effective practice in rural and resource-limited settings. This contribution reinforces the importance of aligning veterinary education with One Health principles and real-world access challenges, preparing professionals who are equipped to work across disciplines and engage meaningfully with communities (Meythaler-Mullins et al.).

The significance of community-governed and culturally grounded partnerships is further advanced in *Evolving culturally competent veterinary care: a community-based partnership with the Santee Nation*. This article describes a long-term, reciprocal collaboration rooted in Indigenous leadership, cultural respect, and shared decision-making. Rather than relying on short-term outreach or externally driven interventions, the partnership emphasizes sustained relationship-building, mutual learning, and culturally responsive pedagogy as pathways to improving veterinary access and trust. The work offers an important model for ethically engaging with Indigenous and historically marginalized communities and demonstrates that equitable access efforts must be co-created with, not delivered to, communities (Orchard, LaPointe et al.).

Innovations in service delivery and capacity building are also central to this Research Topic. The mini-review *Methodologies to assess community-based animal health workers curricula and training programs* examines approaches for evaluating training models that extend animal health services through community-based workers. Such models hold promise for addressing veterinary shortages and access gaps in underserved and remote regions, while strengthening local capacity and sustainability. By emphasizing methodological rigor and program assessment, this work contributes to the growing evidence base needed to responsibly develop and scale alternative workforce models (Matos et al.).

Finally, financial accessibility—one of the most frequently cited obstacles to veterinary care—is addressed in *A prospective observational study of how veterinary clinics and their clients utilized a no-credit-check, third-party managed installment financing option to increase access to veterinary care*. This study provides empirical insight into how alternative financing mechanisms may support care-seeking behavior without relying on traditional credit systems that exclude many clients. By examining real-world clinic and client experiences, the authors contribute practical evidence to ongoing discussions about affordability, ethics, and financial inclusion in veterinary medicine (Cammisa and Hill).

Collectively, the articles in this Research Topic demonstrate that enhancing veterinary access requires coordinated action across disciplines, sectors, and communities. One Health is advanced here not only as a conceptual orientation but as a practical organizing framework for aligning veterinary care with animal welfare professionals, public health, social services, education, and community leadership. The work presented underscores the need to prepare veterinary professionals who can navigate complexity, engage respectfully with diverse populations, and collaborate effectively beyond traditional professional boundaries.

Why does this body of work matter now? Veterinary medicine is increasingly encountering the limits of clinic-centered solutions. The profession is being asked to respond to rising economic precarity, shifting family structures, workforce strain, and widening inequities in ways it was not historically designed to address. The articles collected here reflect a field in transition—one that is beginning to build the partnerships, competencies, and infrastructures required to operate effectively within integrated health and social ecosystems.

This Research Topic moves the conversation from recognition to responsibility. It challenges the field to invest in interprofessional infrastructures, community-led models, financing mechanisms, and educational reforms that make access not a charitable exception but a structural feature of veterinary systems. The insights offered across these contributions provide a foundation for continued scholarship, policy development, and practice innovation aimed at ensuring veterinary care is accessible, inclusive, and responsive to the needs of pet families and the communities who support them.

